# Is there a need of providing at least 3 decimal *P*-value to avoid type 1 error in a clinical research?

**DOI:** 10.1093/icvts/ivac293

**Published:** 2023-02-16

**Authors:** Ashok Kumar, Manisha Nagi, Mukesh Kumar, Maninder Deep Kaur

**Affiliations:** Department of National Institute of Nursing Education, Postgraduate Institute of Medical Education and Research, Chandigarh, India; Department of National Institute of Nursing Education, Postgraduate Institute of Medical Education and Research, Chandigarh, India; Department of Industrial Engineering, Dalhousie University, Halifax Nova, Scotia, Canada; Department of National Institute of Nursing Education, Postgraduate Institute of Medical Education and Research, Chandigarh, India

**Keywords:** P value, Type 1 error, Odds ratio, 95% CI

The original article entitled ‘Density of aortopulmonary collaterals predicts in-hospital outcome in tetralogy of Fallot with pulmonary stenosis’ published by Fang *et al.* in 2021 in your reputed journal. This article is well written and provides evidence for clinicians. The aim of this study was to characterize the anatomy of aortopulmonary collateral (APC) arteries in tetralogy of Fallot and pulmonary stenosis and to determine whether APC density identified on preoperative multidetector cardiac computed tomography predicts in-hospital outcome [1]. I want to congratulate all the authors as they did good job to accomplish the aim of the study. After reading this article thoroughly, I am able to explain more clearly about *P*-value and 95% confidence interval (CI) of odds ratio for multivariable to avoid false-positive results in clinical research.

**Figure 1:**
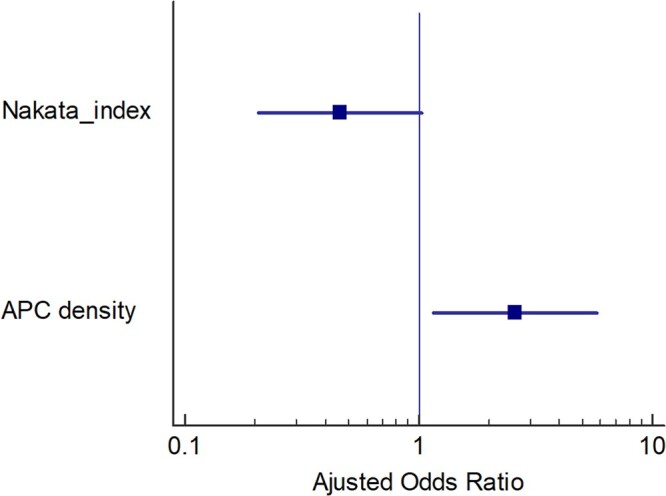
Forest plot showing adjusted odds ratio and their 95% confidence interval for predicting composite outcome. The figure shows that 95% confidence interval of Nakata index is crossing null value of 1, i.e. line of no effect, whereas 95% confidence interval of aortopulmonary collateral density is not crossing null value of 1.

On p. 311, there is subheading of ‘Predictors for the composite outcome’ in which multivariable analysis result was narrated as high APC density [odds ratio 2.585 (1.152–5.800), *P* = 0.02] and low Nakata index [odds ratio: 0.460 (0.206–1.028), *P* = 0.05] as independent predictors for the composite outcome. And also in Table 2 which was headed as ‘Univariable and multivariable logistic regression on predictors associated with composite outcome’ presented the adjusted odds ratio of Nakata index (mm^2^/m^2^) (for each 0.1 mm^2^/m^2^ increase) as adjusted odds ratio: 0.460 (95% CI: 0.206, 1.028), *P*-value = 0.05. On the basis of these results, authors conclude that low Nakata index as an independent predictor for the composite outcome should be re-evaluated by reviewers and by authors as we know that 95% CI of adjusted odds ratio 0.460 of Nakata index was crossing null value of no effect, i.e. 1 (95% CI: 0.206, 1.028) (see Figure 1), and even *P*-value was given in 2 decimals (*P* = 0.05) which might be under power and hinder the true effect. Generally, we give *P*-value in 3 decimals. Various statistical software like SPSS, R, Stata, etc., provide *P*-value or significance value in 3 or >3 decimals to avoid false-positive results or type 1 error that is ‘rejecting the null hypothesis when it is right’ [2]. But on the contrary, in this article, only 2 decimals were given (*P* = 0.05). It might be 0.051 or 0.054 and on rounding off it becomes 0.05 (when researcher provides 2 decimal *P*-value) which might be taken as statistically significant which is not in fact when we look at 95% CI of odds ratio crossing null value of no effect, i.e. 1. It might commit ‘type 1 error’, although it is very low.

It is requested to the esteemed reviewers to get *P*-values in at least 3 decimals for more clarification in clinical research to avoid unintentional type 1 error.
